# Neuroprotective Effect of Hydrogen-Rich Saline against Neurologic Damage and Apoptosis in Early Brain Injury following Subarachnoid Hemorrhage: Possible Role of the Akt/GSK3β Signaling Pathway

**DOI:** 10.1371/journal.pone.0096212

**Published:** 2014-04-24

**Authors:** Yuan Hong, AnWen Shao, Jianfeng Wang, Sheng Chen, HaiJian Wu, Devin W. McBride, Qun Wu, XueJun Sun, JianMin Zhang

**Affiliations:** 1 Department of Neurosurgery, The Second Affiliated Hospital, School of Medicine, Zhejiang University, Hangzhou, China; 2 Department of Neurosurgery, Taizhou Enze Medical Center (Group), Taizhou Hospital, Taizhou, China; 3 Department of Physiology and Pharmacology, Loma Linda University School of Medicine, Loma Linda, California, United States of America; 4 Department of Diving Medicine, The Second Military Medical University, Shanghai, China; St Michael's Hospital, University of Toronto, Canada

## Abstract

**Backgrounds:**

Early brain injury (EBI) plays a key role in the pathogenesis of subarachnoid hemorrhage (SAH). Neuronal apoptosis is involved in the pathological process of EBI. Hydrogen can inhibit neuronal apoptosis and attenuate EBI following SAH. However, the molecular mechanism underlying hydrogen-mediated anti-apoptotic effects in SAH has not been elucidated. In the present study, we aimed to evaluate whether hydrogen alleviates EBI after SAH, specifically neuronal apoptosis, partially via the Akt/GSK3β signaling pathway.

**Methods:**

Sprague-Dawley rats (n = 85) were randomly divided into the following groups: sham group (n = 17), SAH group (n = 17), SAH + saline group (n = 17), SAH + hydrogen-rich saline (HS) group (n = 17) and SAH + HS + Ly294002 (n = 17) group. HS or an equal volume of physiological saline was administered immediately after surgery and repeated 8 hours later. The PI3K inhibitor, Ly294002, was applied to manipulate the proposed pathway. Neurological score and SAH grade were assessed at 24 hours after SAH. Western blot was used for the quantification of Akt, pAkt, GSK3β, pGSK3β, Bcl-2, Bax and cleaved caspase-3 proteins. Neuronal apoptosis was identified by double staining of terminal deoxynucleotidyl transferase mediated nick end labeling (TUNEL) staining and NeuN, and quantified by apoptosis index. Immunohistochemistry and immunofluorescent double-labeling staining was performed to clarify the relationships between neuronal apoptosis and pAkt or pGSK3β.

**Results:**

HS significantly reduced neuronal apoptosis and improved neurological function at 24 hours after SAH. The levels of pAkt and pGSK3β, mainly expressed in neurons, were markedly up-regulated. Additionally, Bcl-2 was significantly increased while Bax and cleaved caspase-3 was decreased by HS treatment. Double staining of pAkt and TUNEL showed few colocalization of pAkt-positive cells and TUNEL-positive cells. The inhibitor of PI3K, Ly294002, suppressed the beneficial effects of HS.

**Conclusions:**

HS could attenuate neuronal apoptosis in EBI and improve the neurofunctional outcome after SAH, partially via the Akt/GSK3β pathway.

## Introduction

Subarachnoid hemorrhage (SAH), typically due to aneurismal rupture, accounts for 2% to 9% of all strokes [1]. However, with the advancement of medical technology and treatment options, there has been little variation in the mortality and morbidity of SAH over the last decade. Traditionally, cerebral vasospasm was thought to be the most important cause of poor outcome in SAH patients. To inhibit vasospasm-induced secondary brain injury, large numbers of experimental and clinical studies have been conducted throughout the world [2]–[4]. Though researches in animal models achieve promising results, however, some large clinical trials shows that the inhibition of vasospasm are not always accompanied with the improvement of outcome following SAH [5], which indicates that not all poor outcomes are vasospasm-dependent, but some other mechanisms might link with the delayed neurologic deficits after SAH. Recently, accumulating evidence shows that early brain injury (EBI), which occurs within the first 72 hours after SAH, plays a pivotal role in the prognosis of SAH. Among all the complex physiology of EBI, neuronal apoptosis has been highlighted [6]. Numerous studies have identified that the severity of neuronal apoptosis is indirectly correlated with neurofunction, which suggests that apoptosis of neurons plays an important role in the quality of life for an SAH survivor [7]–[10].

Oxidative stress, a significant factor of the pathogenesis of SAH-induced EBI, has recently received increased attention for its contribution to the occurrence of apoptosis, the increased production of reactive oxygen species (ROS) and insufficient intrinsic antioxidant enzymes [8], [9], [11], [12]. Accumulating evidence demonstrated that nitrotyrosine, MDA and 8-OHG, which target oxidation of protein, liquid and DNA respectively, increase substantially after SAH [13]–[15] Thus, research has shown that antioxidative agents may improve the outcome of patients with SAH via an anti-apoptotic effect [13], [16]. Hence, pharmacological treatments with antioxidative effects are promising.

Hydrogen, a novel and effective antioxidant, could selectively scavenge the two most aggressive ROS: OH and ONOO-, and there is substantial evidence that hydrogen provides neuroprotection of oxidative stress-induced damage in neurological diseases, such as Parkison’s disease, Alzheimer’s disease, transient and permanent cerebral ischemia and spinal cord injury [17], [18]. Our previous research revealed that hydrogen has a beneficial effect on cerebral vasospasm after SAH [14]. Other similar studies have reported that hydrogen may have therapeutic potential in experimental SAH rats and rabbits, and attenuate EBI by reducing the number of apoptotic cells and brain edema, subsequently improving neurological function [13], [19]. However, the underlying mechanism of hydrogen-mediated inhibition of apoptosis after SAH is still not elucidated.

Serine-threonine kinase (also referred to as protein kinase B), which is downstream of the phosphoinositide 3-kinase (PI3K) pathway, plays a vital role in the cell survival/death pathway [20]. Activation of Akt is dependent on PI3K, since SAH activated PI3K leads to the production of phosphatidylinositol 3,4,5 trisphosphate (PIP3) and phosphatidylinositol 3,4 bisphosphate (PIP2) which are necessary for Akt activation (phosphorylation of Akt at serine-473). Hence, administration of Ly294002, a highly specific PI3K inhibitor, increased neuronal apoptosis by inactivating Akt [21], [22]. Activated Akt promotes neuronal survival mainly by phosphorlation of several downstream macromolecules, such as glycogen synthase kinase 3β (GSK3β), caspase 9 and Bcl-xl/Bcl-2 associated death promoter (BAD) [22], [23]. Akt phosphorylates GSK3β on serine-9 to inactivate it, which prevents neuronal apoptosis. A number of studies have confirmed that the activation of the Akt/GSK3β pathway attenuates apoptosis and correlates with regulation of Bcl-2, Bax and caspase 3 [24], [25] to mediate cell survival in many neurological diseases [26]–[28]. Furthermore, studies revealed that inhibition of oxidative stress by antioxidants could significantly alleviate EBI after SAH via the Akt/GSK3β pathway [22], [29]. These results suggest that hydrogen might attenuate EBI after SAH, specifically neuronal apoptosis, via the Akt/GSK3β signaling pathway. Therefore, in the present study, we sought to test the effect of HS application in an SAH model induced by endovascular perforation.

## Materials and Methods

### Ethics Statement

All experimental protocols involving animals (including all surgical procedure) were approved by the Institutional Animal Care and Use Committee (IACUC) of the Zhejiang University. All rat experimental procedures were performed in accordance with the Regulations for the Administration of Affairs Concerning Experimental Animals approved by the State Council of People’s Republic of China.

### Experimental Animals

Adult male Sprague-Dawley rats weighing 270–330 g were obtained from the Animal Center of Zhejiang Chinese Medical University (Hangzhou, China). Rats were housed in air-filtered units with free access to food and water for 7 days before surgical treatment. The temperature in both the feeding room and the operation room was approximately 25°C.

### HS Production

HS was prepared as described previously [30]. Briefly, H_2_ was dissolved in 0.9% saline for 6 hours under high pressure (0.4 MPa) to a supersaturated level using a HS-producing apparatus (produced by the Diving Medicine Deparment of the Second Military Medical University in Shanghai China). The saturated HS was stored at 4°C at atmospheric pressure in an aluminum bag with no dead volume and sterilized by gamma radiation. To confirm the concentration of hydrogen in the saline, gas chromatography was performed using the method described by Ohsawa et al [16]. HS was freshly prepared every week to ensure a concentration of more than 0.6 mmol/L was maintained.

### SAH Model

SAH was produced by the endovascular perforation method with little modification to previous descriptions [14]. Briefly, animals were anesthetized with an intraperitoneal injection of chloral hydrate (400 mg/kg), and anesthesia was maintained with subsequent injections of chloral hydrate. Rectal temperature was maintained at 37°C during operation with a heating blanket. After a midline skin incision, the left common carotid artery, external carotid artery and internal carotid artery were exposed. The left external carotid artery was ligated and cut, leaving a 3 mm stump. A sharpened 4-0 monofilament nylon suture was then inserted into the left internal carotid artery through the external carotid artery stump to perforate the artery at the bifurcation of the anterior and middle cerebral artery. Sham-operated rats underwent identical procedures except no perforation was performed.

### Experimental Protocol

Eight-five rats were randomly allocated into one of the following groups: (1) sham group (n = 17), (2) SAH group (n = 17), (3) SAH + saline group (n = 17), (4) SAH + HS group (n = 17) and (5) SAH + HS + Ly294002 group (n = 17). All the rats were sacrificed at 24 hours after SAH. Seven rats in each group were perfused with the fixative, the brains were removed for TUNEL staining, immunohistochemistry and immunofluorescent staining. The remaining rats (n = 10/group) were exsanguinated and decapitated, the brains removed and frozen in liquid nitrogen immediately for western blot. Mortality was calculated at 24 hours after SAH.

### Drug Injection

HS (5 mL/kg intraperitoneally) was administered in the SAH + HS and SAH + HS + Ly294002 groups immediately after surgery and repeated 8 hours later. The SAH + saline group received an equal volume of saline at the same time points. The dosage and time points of HS were based on a previous study [30].

To investigate the role of the PI3K pathway after SAH, we administered Ly294002 (2-(4-morpholinyl)-8-phenyl-4H-1-benzopyran-4-one) (Cell Signaling Technology, USA), which is a highly selective inhibitor of PI3K. Anesthetized rats were positioned in a stereotaxic frame, and Ly294002 (50 mmol in 25% dimethyl sulfoxide and PBS) was injected intracerebroventricularly (10 µL, bregma; 1.4 mm lateral, 0.8 mm posterior, 3.6 mm deep) with a syringe pump 30 minutes before SAH.

### Assessment of Neurological Score

At 24 hours post-SAH, animals in each group were recorded for neurological scores with the modified Garcia’s method by an individual who was blinded to the experimental groups [31]. An 18-point system was used to assess animals’ neurological deficits from six aspects, including spontaneous activity (0–3), symmetry movements of all limbs (0–3), outstretching of forelimbs (0–3), climbing (1–3), body proprioception (1–3), and response to vibrissae touch (1–3). A minimum scores at least 3 and a maximum scores 18. A lower score represents a more severe neurological deficit.

### Assessment of SAH Grade

SAH grade was obtained by using a previously described grading system [32]. Briefly, the basal cistern was divided into six segments, grading of each segment based on subarachnoid blood blot as follows: 1. No subarachnoid blood (scores = 0); 2. Minimal subarachnoid blot (scores = 1); 3. Moderate subarachnoid blot with recognizable arteries (scores = 2); 4. Blood blot covering all arteries (scores = 3). The minimum neurological score is 0 and the maximum is 18, which means that the SAH grade was in a total score from 0 to 18. All tests were conducted by an individual who was blinded to the experimental groups. Only animals experiencing severity score >8 were included in this work.

### Double Staining of TUNEL and NeuN, pAkt (Ser-473)

Double label staining of TUNEL with neuron-specific nuclear protein (NeuN) was performed to explore colocalization of apoptosis cells and neurons. Briefly, the rats were perfused with 4% paraformaldehyde and postfixed by immersion in the same fixative overnight. Then the brains were paraffin-embedded, and sectioned at a thickness of 10 µm. TUNEL staining was performed according to the manufacturer’s protocol of the commercial kit (Roche, Switzerland), followed by antibody staining against NeuN (1∶200, Abcam, UK). Finally, the sections were covered with 4′6-diamidino-2-phenylindole (DAPI) (Beyotime, China) and observed under a fluorescence microscope (Leica, Germany).

Double staining of pAkt (Ser-473) with TUNEL staining was performed to clarify the relationship between apoptosis and pAkt (Ser-473). The sections were stained with the antibody against pAkt (serine-473) (1∶200, Cell signaling Technology, USA), followed by TUNEL staining. Then the sections were covered with DAPI (Beyotime, China) and observed with a fluorescence microscope (Leica, Germany).

### Western Blot Analysis

Rats were decapitated at 24 hours after SAH induction or sham injury. The same part of left cortical samples were obtained and whole cell protein extraction was performed as previously described [14], [29]. Briefly, the samples were homogenized and centrifuged at 12,000 g for 10 minutes at 4°C. Supernatants were collected, and protein concentrations were determined by using a BCA kit (Beyotime, China). The protein samples were separated by 10–15% sodium dodecyl sulfate–polyacrylamide gel electrophoresis and transferred to polyvinylidene fluoride membranes (Millipore, USA). After blocking with 5% nonfat dry milk in TBS for 2 hours, the membranes were incubated overnight at 4°C with primary antibodies as following: anti-pAkt (serine-473) (1∶1000, Cell signaling Technology, USA), anti-Akt (1∶1000, Cell signaling Technology, USA), anti-pGSK3β (serine-9) (1∶1000, Cell signaling Technology, USA), anti-GSK3β (1∶1000, Cell signaling Technology, USA), anti-Bax (1∶1000, Santa Cruz, USA), anti-Bcl-2 (1∶1000, Santa Cruz, USA), anti-cleaved caspase-3 (1∶500, Santa Cruz, USA), and anti-β-actin (1∶2000, Abcam, UK). After incubation, the membranes were washed with TBST and incubated with horseradish-peroxidase conjugated secondary antibodies for 2 hours at room temperature. We detected the antigen–antibody complexes using an ECL Plus chemiluminescence reagent kit (Beyotime, China) and exposed them to x-ray film.

### Immunohistochemistry

We performed immunohistochemistry staining according to the manufacturer's protocol of the kit. Rats were anesthetized and sacrificed by perfusion with 4% paraformaldehyde 24 hours after SAH induction or sham injury. The sections were incubated with the antibodies against pAkt (serine-473) (1∶1000, Cell signaling Technology, USA) and pGSK3β (serine-9) (1∶1000, Cell signaling Technology, USA).

### Immunofluorescent Double-labeling Staining

To evaluate colocalization of neuron and pAkt (Ser-473) or pGSK3β (Ser-9), we performed double staining for NeuN and pAkt (Ser-473) or pGSK3β (Ser-9) using the following antibodies: anti-pAkt (serine-473) (1∶200, Cell signaling Technology, USA) or anti-pGSK3β (Ser-9) (1∶200, Cell signaling Technology, USA), with Texas Red–conjugated immunoglobulin G, and anti–NeuN antibody (1∶200, Abcam, UK). Subsequently, the sections were covered with DAPI (Beyotime, China) and observed with a fluorescence microscope (Leica, Germany).

### Quantification and Statistical Analysis

Data were expressed as means ± standard deviation (SD). One-way analysis of variance (ANOVA) was used to compare means of different groups. If the null hypothesis of no difference between groups was rejected, a Tukey’s test was performed for post-hoc comparison. Fisher exact test was used in two group comparisons for mortality analysis. All statistical values were calculated using SPSS 16.0. Statistically significance was accepted with *P*<0.05.

## Results

### Mortality and SAH Grade

**Figure 1:**
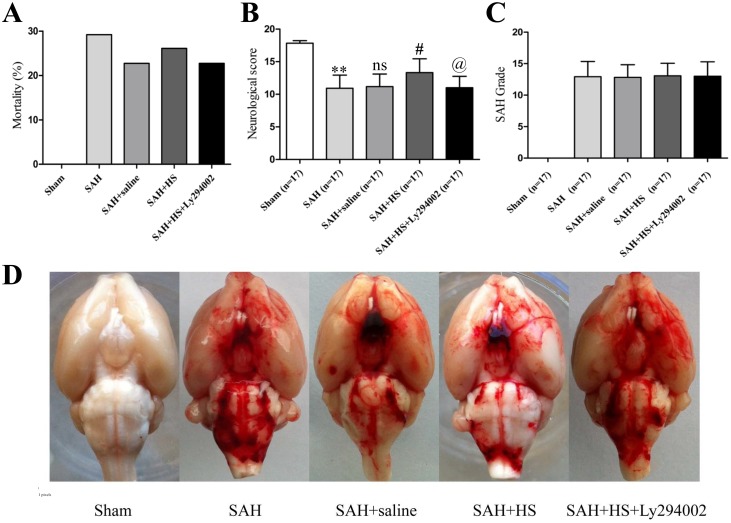
The mortality, neurological score, and SAH grade among each group. A. SAH group was 35% (9 of 26 rats), SAH + saline group was 29% (7 of 24 rats), SAH + HS group was 29% (7 of 24 rats), and SAH + HS + Ly294002 group was 26% (6 of 23 rats). No significant differences were noted in mortality among each group. B. SAH significantly impaired neurological function (*P*<0.01) at 24 hours compared with the sham rats, whereas HS-treated animals had significantly improved neurological scores compared with the SAH rats (*P*<0.05). However, Ly294002 abolished the beneficial effect of HS on neurobehavioral function (*P*<0.05). C. The SAH grade scores had no significant difference among all groups: 12.9±1.4 in SAH group, 12.8±2.0 in SAH + saline group, 13.1±2.0 in SAH + HS group, 13.0±2.3 in SAH + HS + Ly294002 group (*P*>0.05). D. Subarachnoid blood clots were mainly found around the circle of Willis and ventral brainstem. Similar severity of SAH bleeding was obtained in all groups 24 hours after SAH. **P<0.01 vs. sham group; ns *P*>0.05 vs. SAH group; # *P*<0.05 vs. SAH + saline group; @ *P*<0.05 vs. SAH + HS group.

No significant differences were observed in body weight or body temperature among groups. No rats died in the sham group (0 of 17 rats). About 30% rats (29 of 97) assigned to the verum group died within 24 hours after SAH induction. No statistical significance was observed for mortality between operated groups: SAH group was 35% (9 of 26 rats), SAH + saline group was 29% (7 of 24 rats), SAH + HS group was 29% (7 of 24 rats), and SAH + HS + Ly294002 group was 26% (6 of 23 rats) (Fig. 1A).

At 24 hours after SAH, subarachnoid blood clots were mainly found around the circle of Willis and ventral brainstem (Fig. 1D). The SAH grade scores had no significant difference among all groups: 12.9±1.4 in the SAH group, 12.8±2.0 in the SAH + saline group, 13.1±2.0 in the SAH + HS group, 13.0±2.3 in the SAH + HS + Ly294002 group (*P*>0.05; Fig. 1C). HS had no effect on bleeding compared with the SAH groups. Twenty SAH rats were excluded from this study because of mild bleeding (SAH grade score≤8).

### HS Attenuated Neurologic Deficits

SAH significantly impaired neurological function (*P*<0.01) at 24 hours compared with the sham rats, whereas HS-treated animals had significantly improved neurological scores compared with the SAH rats (*P*<0.05). However, Ly294002 abolished the beneficial effect of HS on neurobehavioral function (*P*<0.05; Fig. 1B).

### HS Alleviated Neuronal Apoptosis in the Cortex at 24 Hours after SAH

**Figure 2:**
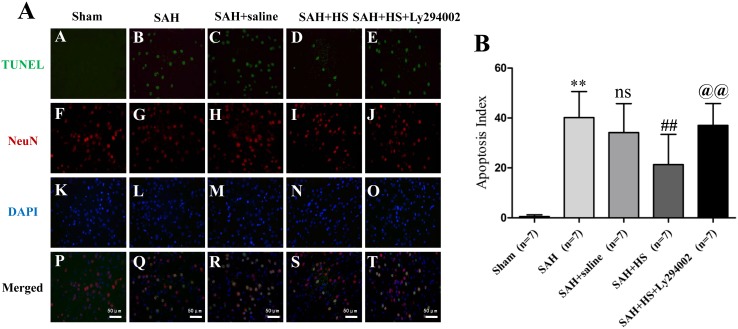
Representative double immunofluorescence staining for TUNEL (green) and NeuN (red) at 24 hours after SAH. Double immunofluorescence staining (A) and quantitative analysis (B) showed that almost no TUNEL-positive apoptotic neurons were detected in the sham group. In the SAH and SAH + saline groups, the apoptotic cells increased markedly compared with the sham group (*P*<0.01), and mainly colocalized with neurons. However, compared with the SAH or SAH + saline groups, administration of HS substantially reduced the number of apoptotic neurons (*P*<0.01), which was reversed by Ly294002 (*P*<0.01). DAPI (blue) as a nuclear marker. Scale bar 50 mm. **P<0.01 vs. sham group; ns *P*>0.05 vs. SAH group; ## *P*<0.01 vs. SAH + saline group; @@ *P*<0.01 vs. SAH + HS group.

Fig. 2 shows almost no TUNEL-positive apoptotic neurons were detected in the sham group. In the SAH and SAH + saline groups, the apoptotic cells increased markedly compared with the sham group (*P*<0.01), and mainly colocalized with neurons. However, compared with the SAH or SAH + saline group, administration of HS substantially reduced the number of apoptotic neurons (*P*<0.01), which was reversed by Ly294002 (*P*<0.01; Fig. 2).

### HS Reduced the Expression of Bax and Cleaved Caspase-3 Proteins, Increased the Expression of Bcl-2 in the Left Cortex at 24 Hours after SAH

**Figure 3:**
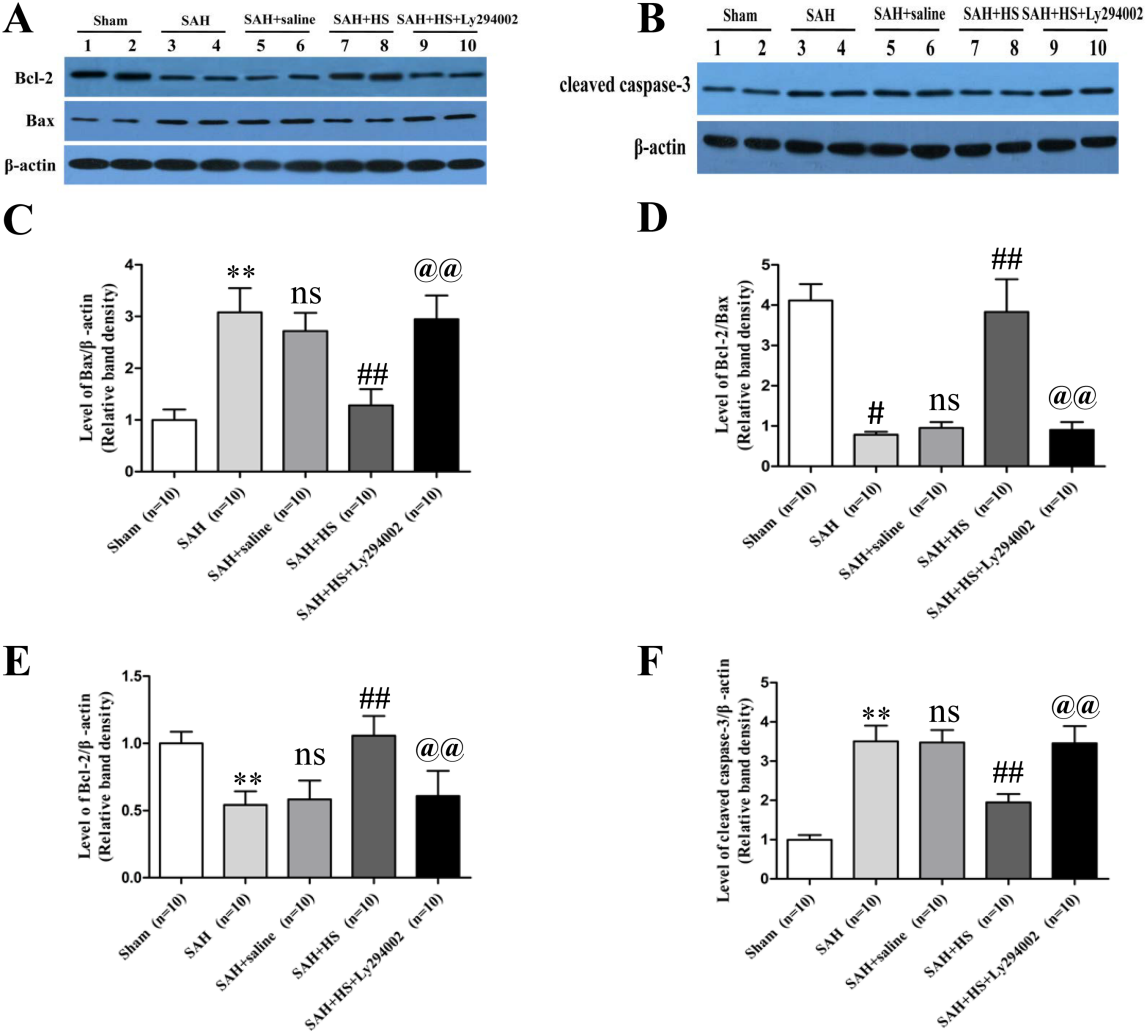
Representative western blots and quantitative analysis of Bcl-2, Bax and cleaved caspase-3 in the left cortex at 24 hours after SAH. The Bcl-2 protein level was significantly up-regulated by HS administration compared with the SAH and SAH + saline groups, which was down-regulated by Ly294002 (*P*<0.01; Fig. 3A, 3C). The expression of Bax protein in the HS groups showed a markedly decreased level compared with the SAH and SAH + saline groups, which was inhibited by Ly294002 (*P*<0.01; Fig. 3A, 3D). Moreover, the ratio of Bcl-2 and Bax protein was increased after HS treatment compared with the SAH and SAH + saline groups, and was abolished by Ly294002 (*P*<0.01; Fig. 3E). The expression of cleaved caspase-3 protein shows a similar trend as the Bax protein (**P**<0.01; Fig. 3B, 3F). **P<0.01 vs. sham group; ns *P*>0.05 vs. SAH group; ## *P*<0.01 vs. SAH + saline group; @@ *P*<0.01 vs. SAH + HS group.

The Bcl-2 protein level was substantially decreased in the SAH and SAH + saline groups compared with the sham group at 24 hours after SAH (*P*<0.01). However, the Bcl-2 protein level was significantly up-regulated by HS administration compared with the SAH and SAH + saline groups which was down-regulated by Ly294002 (*P*<0.01; Fig. 3A, 3D). The expression of Bax protein in the HS groups showed a markedly decreased level compared with the SAH and SAH + saline groups (*P*<0.01), which was inhibited by Ly294002 (*P*<0.01; Fig. 3A, 3C). Moreover, the ratio of Bcl-2 and Bax protein was increased after HS treatment compared with the SAH and SAH + saline groups (*P*<0.01), and was abolished by Ly294002 (*P*<0.01; Fig. 3E). The expression of cleaved caspase-3 protein shows a similar trend as the Bax protein (*P*<0.01; Fig. 3B, 3F).

### HS Enhanced the pAkt Activation and pGSK3β Expression in the Left Cortex at 24 Hours after SAH

**Figure 4:**
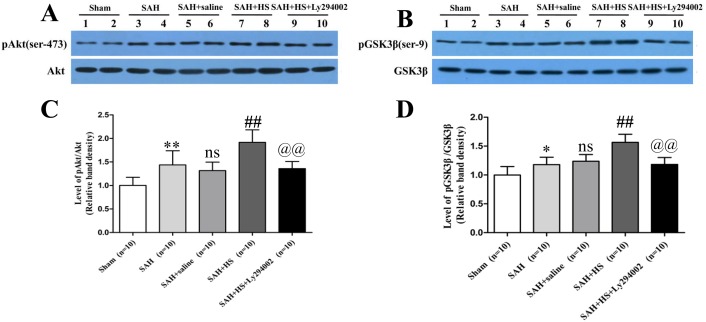
Representative western blots and quantitative analysis of pAkt (ser-473) and pGSK3β (serine-9) in the left cortex at 24 hours after SAH. The levels of pAkt and pGSK3β were low in the sham group (Fig. 4), which was significantly increased after administration of HS compared with the SAH and SAH + saline groups (*P*<0.01). Ly294002 inhibited the increased expression of pAkt and pGSK3β (*P*<0.01). The total Akt and total GSK3β expression was similar among all groups. *P<0.05 vs. sham group; **P<0.01 vs. sham group; ns *P*>0.05 vs. SAH group; ## *P*<0.01 vs. SAH + saline group; @@ *P*<0.01 vs. SAH + HS group.

The protein levels of pAkt and pGSK3β were low in the sham group, which was significantly increased after administration of HS compared with the SAH and SAH + saline groups (*P*<0.01). Ly294002 inhibited the increased expression of pAkt and pGSK3β (*P*<0.01; Fig. 4). The total Akt and total GSK3β expression was similar among all groups, indicating that treatment with HS could enhance the expression of pAkt and pGSK3β.

**Figure 5:**
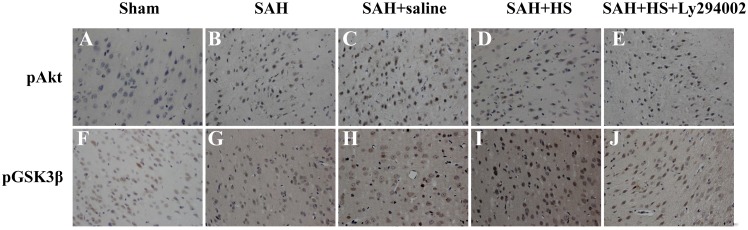
Representative immunohistochemical staining for pAkt (ser-473) and pGSK3β (serine-9) in the left cortex at 24 hours after SAH. The immunohistochemical staining for pAkt (serine-473) and pGSK3β (serine-9) have similar trends. pAkt (serine-473) and pGSK3 (serine-9) in the sham group have only a slight immunoactivity. However, after administration of HS, the immunoactivity of pAkt (serine-473) and pGSK3 (serine-9) are significantly higher than the SAH and SAH + saline groups. In contrast to the SAH + HS group, the pAkt and pGSK3β positive neurons decreased in the SAH + HS + Ly294002 group. These results suggests that HS treatment could induce phosphorylation of Akt and GSK3β following experimental SAH, and when Ly294002 is administered, the phosphorylation of Akt and GSK3β is significantly suppressed.

Immunohistochemical staining for pAkt (serine-473) and pGSK3β (serine-9) shows similar trends (Fig. 5). pAkt (serine-473) and pGSK3 (serine-9) in the sham group have only a slight immunoactivity. However, after administration of HS, the immunoactivity of pAkt (serine-473) and pGSK3 (serine-9) are significantly higher than the SAH and SAH + saline groups. In contrast to the SAH + HS group, the number of pAkt and pGSK3β positive neurons decreased markedly in the SAH + HS + Ly294002 group. These results suggests that HS treatment could induce phosphorylation of Akt and GSK3β following experimental SAH, and when Ly294002 is administered, the phosphorylation of Akt and GSK3β is significantly suppressed.

### pAkt–positive and GSK3β-positive Cells Colocalized Mainly with Neurons in the Cortex at 24 Hours after SAH

**Figure 6:**
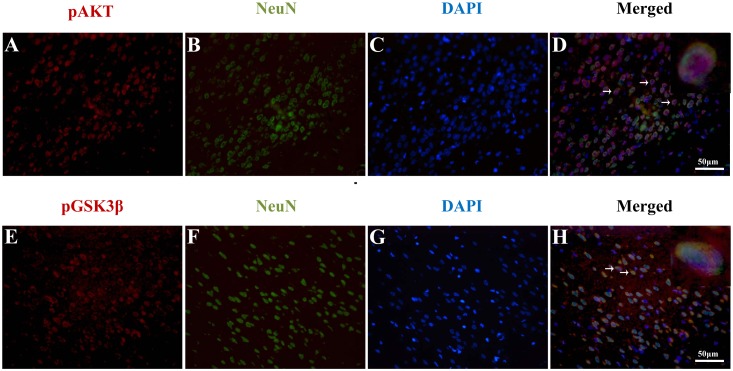
Representative double immunofluorescence staining for pAkt (ser-473) and NeuN, pGSK3β (serine-9) and NeuN at 24 hours after SAH. pAkt-positive cells and pGSK3β-positive cells colocalized mainly with neurons in the cortex 24 hours after SAH respectively (Fig. 6D, 6H). DAPI (blue) as a nuclear marker. Scale bar 50 mm.

To investigate whether activation of Akt/GSK3β signaling occurs mainly in neurons, double immunofluorescent for pAkt (serine-473) and NeuN, and pGSK3β (serine-9) and NeuN was performed. We found pAkt-positive cells and pGSK3β-positive cells colocalized mainly with neurons in the cortex at 24 hours after SAH (Fig. 6). These results indicated that phosphorylation of both Akt and GSK3β occurred mainly in neurons after SAH.

### Double Immunofluorescence Staining Analysis for pAkt (Serine-473) and TUNEL in the Cortex at 24 Hours after SAH

**Figure 7:**
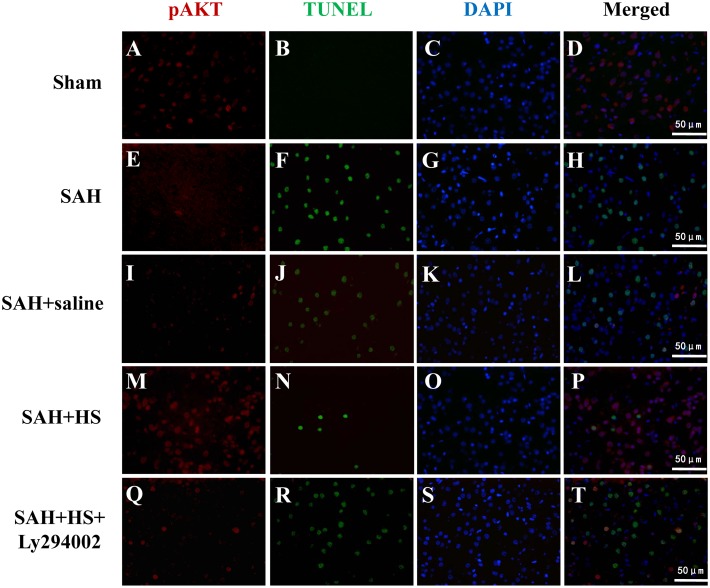
Representative double immunofluorescence staining for phospho-Akt (ser-473) (red) and TUNEL (green) at 24 hours after SAH. TUNEL-positive cells spread throughout the cerebral cortex, but these cells mostly did not colocalize with pAkt–positive cells. TUNEL-positive cells significantly decreased after administration of HS, while pAkt-positive cells markedly increased. However, few colocalization was observed between pAkt-positive cells and TUNEL-positive cells. Scale bar 50 mm.

To detect the distribution of pAkt-positive cells and apoptotic cells, double immunofluorescent for pAkt (serine-473) and TUNEL staining was performed. 24 hours after SAH, TUNEL-positive cells spread throughout the cerebral cortex, but these cells mostly did not colocalize with pAkt–positive cells (Fig. 7). TUNEL-positive cells significantly decreased after administration of HS, while pAkt-positive cells markedly increased. However, few colocalization was observed between pAkt-positive cells and TUNEL-positive cells. These results suggest that the cellular distribution of pAkt (serine-473) is different from that of apoptotic cells after SAH.

## Discussion

In the present study, we investigated whether HS treatment induced neuroprotection is mediated, at least, partially via the Akt/GSK3β pathway in an animal model of SAH. Firstly, the endovascular perforation model of SAH induced significant neuronal apoptosis. HS significantly reduced the percentage of neuronal apoptosis and improved neurological function. In addition, pAkt and pGSK3β express mainly in neurons, and after administration of HS, pAkt and pGSK3β were markedly up-regulated. Furthermore, hydrogen, as a novel antioxidant, remarkably increased expression of Bcl-2 and decreased the level of Bax and caspase 3. Finally, the inhibitor of PI3K, Ly294002, significantly suppressed the favorable effects of HS. Our results showed that HS could attenuate neurologic damage and apoptosis in EBI after SAH, specifically via the Akt/GSK3β signaling pathway.

After SAH, appearance of mitochondria dysfunction leads to subsequently improving oxidative stress. According to literature, the main cause of oxidative stress following SAH is oxyhaemoglobin release from erythrocytes in the subarachnoid blood clot [33], [34]. The increased level of oxidative stress, which occurs in different brain cells including neurons, is accompanied by neuronal apoptosis after SAH [8], [35], [36]. Therefore, inhibiting oxidative stress-induced neuronal apoptosis is a critical intervention strategy in SAH, and a line of evidence demonstrated that many antioxidant treatment reduced apoptosis of neurons after SAH [13], [16], [29]. Consistent with previous studies, we found neuronal apoptosis increased significantly after SAH induction, and decreased markedly after HS treatment, which was reversed by Ly294002. These results suggested that HS, as a specific antioxidant, is effective in attenuating neuronal cell death post-SAH.

Oxidative stress increases PI3K reaction products, which moderately triggers Akt phosphorylation within several cell types and animal models [29], [37], [38], but excessive oxidative stress, such as after SAH, may lead to dephosphorylation of Akt, followed by apoptosis [29]. Akt is activated by phosphorylation at the serine 473 residue, subsequently leading to phosphorylation of its downstream target GSK3β at serine 9. As a signaling pathway which regulates cell survival, the Akt/GSK3β pathway has been implicated in many diseases including nervous system diseases, such as global and focal cerebral ischemia, traumatic brain injury, and spinal cord injury [26], [27], [39]. The importance of the Akt/GSK3β pathway in SAH was first reported by Hidenori Endo et al., followed by several series of studies concerning the Akt/GSK3β pathway, revealing that this pathway plays an important role in regulating neuronal apoptosis [22], [29]. Previously also reported, the phosphorylation of Akt and GSK3β mainly occurs in neurons. Our present study demonstrated that activation of Akt and GSK3β enhanced after SAH onset, administration of HS improved activation of Akt and GSK3β, resultantly improving neuronal survival after SAH. Moreover, an inhibitor of PI3K, Ly294002 prevented phosphorylation of Akt and GSK3β, subsequently leading to increased cell death. In addition, immunofluorescence studies revealed that activation of Akt and GSK3β occurred mainly within neurons, pAkt and pGSK3β-positive cells rarely colocalized with TUNEL-positive cells. These results proved that the hydrogen-triggered neuroprotective effect is, at least partially, associated with anti-apoptosis following SAH by activation of the Akt/GSK3β pathway in neurons.

To further explore the mechanism of anti-apoptosis by hydrogen, a few apoptosis proteins were measured: Bcl-2, Bax and caspase-3. Previous studies have shown that the balance of Bcl-2 and Bax has a tight connection with cell death and survival [40]–[42]. Caspase-3 also plays a key role in the caspase-independent apoptosis pathway, taking part in diseases such as subarachnoid hemorrhage, spinal cord injury, and cerebral ischemia [19], [43], [44]. As a familiar pro-apoptotic member of the Bcl-2 family, Bax level elevated dramatically after SAH [45], leading to Bax-mediated mitochondrial membranes permeabilization, which causing release of cytochrome c from mitochondria to cytosol. In the cytosol, apoptosome, assembly of cytochrome c, Apaf-1 and procaspase-9, is significant for the caspase-9 activation. which in turn cleaves caspase-3, leading to neuronal apoptosis [46]. Similarly in our experiment, we observed increased expression of Bax and caspase-3, and decreased expressions of Bcl-2 after SAH, but injection of HS reverses these protein expressions. Thus, hydrogen seems to activate the Akt/Gsk3β pathway and reduce neuronal apoptosis via regulation of the Bcl-2 family and caspase-3.

**Figure 8:**
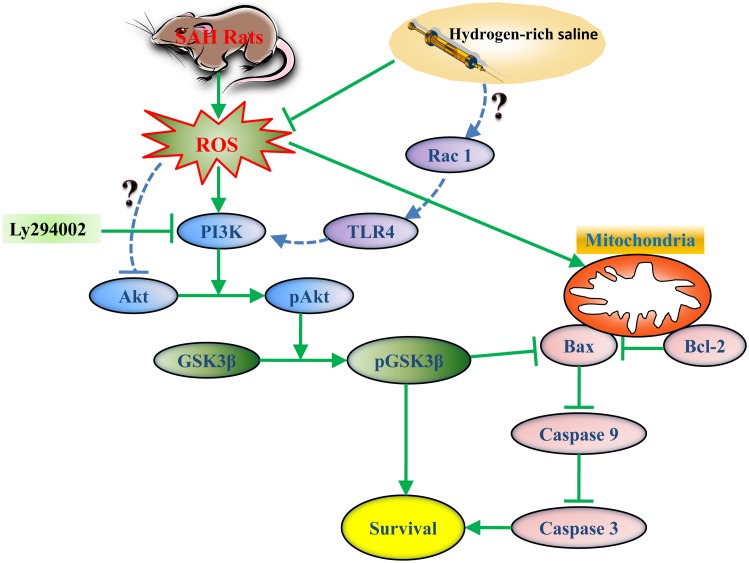
The potential involved molecular mechanism underlying hydrogen-rich saline-mediated anti-apoptotic effect.

The activation of Akt by hydrogen still remains unclear. Previous research, as well as the results of this study, speculates that the process may be due to the following factors (Fig. 8): (1) Excessive oxidative stress contributes to Akt dephosphorylation. Hydrogen could markedly eliminate SAH-induced oxidative stress resulting in a decreased dephosphorylation of Akt. (2) TLR4 could increase activation of PI3K via activation of Rac1. Previous studies have reported that hydrogen can exert its anti-inflammatory effect by regulating the TLR4-signaling pathway [47]. While in our study we observed hydrogen mediated effects via the Akt/GSK3β pathway, hydrogen may also activate Akt via the activation of TLR4. These hypotheses will be the focus of future studies.

In the present study, we explored the role of Akt/GSK3β signaling pathway in the HS-induced neuroprotection after SAH. However, we could not conclude that Akt/GSK3β pathway is the only signaling pathway involved in the beneficial effect of HS. Most published articles showed that hydrogen has significant effect in treating various diseases, mostly due to its anti-oxdative activity [14], [17]–[19]. However, oxidative stress after SAH could impact on different signal pathwaya, including extracellular signal-regulated protein kinase (ERK) 1/2 pathway, NF-κB pathway, c-Jun NH2-terminal kinase (JNK) pathway et al, and previous researches have shown that hydrogen could alleviate oxidative stress-induced injury targeting these pathways mentioned above [48]–[50]. Therefore, further studies focused on exploring other signaling pathway, which may take part in the underlying mechanism of HS therapeutic effect in SAH will be meaningful.

However, this study has several limitations. Firstly, only the tissue in cerebral cortex is analyzed, however, other regions of the brain, such as the hippocampus, are likely to have increased neuronal apoptosis in EBI following SAH. Thus the activation of the Akt/GSK3β pathway by hydrogen may also occur in other brain regions. Additionally, Only one dose and one treatment regime was investigated. The therapeutic time window, the optimal dosage of HS treatment in SAH needs to be addressed in our future studies. Furthermore, the molecular basis underlying Akt activation mechanism induced by HS is still obscure. Our further study will focus on illustrating this issue.

## Conclusion

Based on the results of the present study, the Akt/GSK3β pathway at least partially mediates HS-induced neuroprotection against EBI after experimental SAH. This is the first study to investigate the mechanism of hydrogen-induced anti-apoptotic effect on EBI after SAH, and, according to the findings, HS may be an effective therapeutic for SAH-induced EBI. We argue that the therapeutic effect of hydrogen in SAH patients warrants further clinical trials.
