# Expression of Concern: Neuroprotective effect of hydrogen-rich saline against neurologic damage and apoptosis in early brain injury following subarachnoid hemorrhage: Possible role of the Akt/GSK3β signaling pathway

**DOI:** 10.1371/journal.pone.0344335

**Published:** 2026-03-09

**Authors:** The PLOS One Editors

After this article [1] was published, concerns were raised regarding results presented in Figs 1, 3, 6 and 7, and compliance with the PLOS Animal Research policy. Specifically,

- Fig 1D in [1] appears similar to Fig 2A in [2, retracted in 3], and to Fig 3A in [4].
- Fig 6 appears similar to Fig 3A in [2, retracted in 3].
- Fig 7C (DAPI, Sham) appears similar to Fig 7O (DAPI, SAH+HS).
- The ethics statement does not report an ethics approval number or date. Additionally, the use of chloral hydrate for surgical anesthesia may not fully align with internationally accepted standards at the time of publication.

The first author AS stated that Fig 7C was wrongly placed, but that the associated merged panel (Fig 7D) is correct. They provided an updated Fig 7 with a corrected panel C, which has been included with this notice as S1 File. The underlying images for this figure were not provided upon editorial request.

The authors did not respond to editorial requests for comment on the remainder of the concerns.

The *PLOS One* Editors issue this Expression of Concern to notify readers of the above concerns.

## Supporting information

S1 File Updated Figure 7. (TIF)
